# Acute Promyelocytic Leukemia

**DOI:** 10.3390/cancers12123718

**Published:** 2020-12-11

**Authors:** Xavier Thomas, Maël Heiblig

**Affiliations:** Department of Hematology, Lyon-Sud Hospital, Hospices Civils de Lyon, 69495 Pierre-Bénite, France

Acute promyelocytic leukemia (APL) is a distinct subtype of acute myeloid leukemia (AML) cytogenetically characterized by a balanced reciprocal translocation between chromosomes 15 and 17, which results in the fusion between the promyelocytic leukemia (*PML*) gene and retinoic acid receptor-α (*RARα*). Due to a severe bleeding tendency often resulting in an early fatal course, APL has historically been considered as one of the most fatal forms of AML. Advances in therapy, including anthracycline-based chemotherapy, has significantly improved the outcome. Additionally, the introduction of all-*trans* retinoic acid (ATRA) and the development of arsenic trioxide (ATO)-containing regimens has transformed APL into the most curable form of AML in adults. Treatment with those agents introduced the concept of cure through targeted therapy. With the revolutionary ATRA–ATO combination therapies, chemotherapy may now safely be omitted, at least in low-risk APL patients. Concomitantly, expert panels have recommended that molecular remission should be considered a therapeutic objective in APL, and molecular response has been adopted as a study endpoint in modern clinical trials.

Despite the fact that APL is one of the most characterized form of AML and constant advances have been made in its treatment, open issues remain. The series of eight articles (four original articles and four reviews), presented by international leaders in the field of biology and treatment of APL, in this special issue of *Cancers*, approaches major issues among areas of ongoing needs, such as a better understanding of relationships between genetic events involved in APL, of APL-like diseases, of blast characteristics of prognostic value, of mechanisms and prevention of the differentiation disease favored by ATRA and/or ATO therapy, and of decision-making in specific situations such as APL arising during pregnancy.

The molecular mechanisms involved in its development and progression are still a matter of study. Although the *PML-RARα* rearrangement is the cytogenetic hallmark of APL, it is not alone able to trigger the whole leukemic phenotype. Secondary cooperating genetic events accumulated over time are essential to ultimately trigger the whole leukemic phenotype. In this special issue of *Cancers*, several papers report those secondary cooperating events [1,2]. Analyses by next-generation sequencing (NGS) approaches helped to determine molecular profiles defined by recurrent alterations in genes associated with signaling pathways (*FLT3*, *NRAS*, *KRAS*), tumor suppression (*WT1*), chromatin organization (*AR1D1B*, *AR1D1A*), oncogenes (*SALL4*, *MED12*, *NSD1*), and rarer mutations in other pathways, including *NPM1* mutations, DNA methylation (*DNMT3A*, *IDH1/2*, *TET2*), or epigenetic regulation (*ASXL1*). A meta-analysis showed that APL patients with *FLT3-ITD* or *FLT3-D835* (the most frequent co-occurring events to *PML-RARα*) are more likely to present high white blood cell counts and have a poorer prognosis than those without these mutations [2]. However, uncommon mutations in epigenetic modifier genes (*DNMT3A*, *IDH1/2*, *TET2*, *ASXL1*) have been associated with a poor prognosis in terms of overall survival and disease-free survival [1]. A mutation spectrum, involving *FLT3*, *WT1*, *NRAS*, *KRAS*, *ID1*, *BAALC*, *ERG*, *KMT2E*, might enable the prediction of clinical outcomes and the categorization of APL patients in different risk subsets [1]. An integrative score in APL, combining gene mutations with expression analysis, has therefore been proposed, categorizing patients in different sets with differences in terms of response rate, relapse, and survival [1]. The clinical relevance of the *SLIT2* gene, an embryonic gene from the SLIT-ROBO family, was reported in an international study [3]. Reduced *SLIT2* expression was associated with high leukocyte counts and reduced overall survival. Blasts with *SLIT2 ^high^* transcript levels were associated with cell cycle arrest, while *SLIT2 ^low^* blasts displayed a more stem-cell like phenotype [3]. This study suggests that the tumor suppressive function of SLIT2 could be considered as a prognostic marker in APL.

If most APL are caused by the translocation of *PML-RARα*, several other types of fusions leading to variant rearrangements of the *RARα* fusion gene have been described over the years. Two reviews published in this *Cancers* issue put in perspective the recent literature on the current understanding of genetic, pathogenesis and therapy response for these APL-like diseases, stressing the role of global deregulated retinoic acid signaling in their pathogenesis [4,5]. Strong recommendations on the appropriate management of those variant forms were not possible due to the low number (less than 1% of APLs) and heterogeneity of reported patients [5]. However, several mechanisms of action were proposed and the existence of mutations of *RARα*, independently from their fusions, was recognized in other conditions leading to the potential implication of ATRA and to a lesser extend ATO in the treatment of multiple cancer types [4].

Several analyses have established a relationship between an unfavorable outcome and several characteristics, including older age, variant chromosomal abnormalities, phenotypic features, *FLT3* mutations, and presence of Bcr3 *PML-RARα* isoforms. However, these observations have not received approval to amend the standard therapy for APL. In an original article of this special issue on APL, the Japan Adult Leukemia Study Group (JALSG) analyzed the data of the 344 patients enrolled in the APL204 trial prospectively treated with ATRA combined with chemotherapy, followed by maintenance therapy using ATRA or tamibarotene, in order to identify important prognostic factors [6]. In multivariate analyses, overexpression of CD56 in blast appeared as an independent unfavorable prognostic factor for relapse-free survival. This tends to confirm the PETHEMA-LPA2012 study which included intensified consolidation for CD56^+^ patients, and suggests the assessment of quantitative change of CD56 during and after treatment by an advanced multicolor flow cytometry.

Differentiation syndrome, which develops in approximately 5–25% of APL patients, is a life-threatening condition triggered by a release of inflammatory cytokines and chemokines by blastic cells; these differentiate in response to ATRA and/or ATO therapy. An original article of this special issue demonstrates that atypical expression of the enzyme transglutaminase 2 (TG2) leads to the generation of inflammation, and could serve as a potential target for the prevention of differentiation syndrome [7].

APL during pregnancy is a challenging situation, which may severely complicate the management of pregnancy, labor, and delivery. An original paper of this special issue analyzed the results of the main cases reported from the literature [8]. Complete response rate remained high despite spontaneous and induced abortion experienced in women diagnosed during the first trimester. Gestational age did not seem to affect the outcomes in the mother, but was closely related to fetal viability. Despite a lack of teratogenic effects reported in neonates, the use of potential teratogenic agents, such as ATRA and ATO, should be done judiciously according to gestational age.

APL remains the best example of how targeted therapy can trigger definitive cures and have paved the way in which cancer should be treated. However, open issues still remain. This special issue of *Cancers* represents a collaborative and international effort that aims to develop better understanding in APL by blending articles regarding genetic, prognostic, and therapeutic approaches. The articles collectively highlight future prospects for improving again therapy and recall the constant progresses made over the last decades yielding APL status to evolve from highly fatal to highly curable.
